# Disruption of the Gene Encoding Endo-β-1, 4-Xylanase Affects the Growth and Virulence of *Sclerotinia sclerotiorum*

**DOI:** 10.3389/fmicb.2016.01787

**Published:** 2016-11-10

**Authors:** Yang Yu, Jifen Xiao, Jiao Du, Yuheng Yang, Chaowei Bi, Ling Qing

**Affiliations:** College of Plant Protection, Southwest UniversityChongqing, China

**Keywords:** *Sclerotinia sclerotiorum*, endo-β-1, 4-xylanase, sclerotia, carpogenic germination, virulence, gene deletion

## Abstract

*Sclerotinia sclerotiorum* (Lib.) de Bary is a devastating fungal pathogen with worldwide distribution. *S. sclerotiorum* is a necrotrophic fungus that secretes many cell wall-degrading enzymes (CWDEs) that destroy plant’s cell-wall components. Functional analyses of the genes that encode CWDEs will help explain the mechanisms of growth and pathogenicity of *S. sclerotiorum*. Here, we isolated and characterized a gene *SsXyl1* that encoded an endo-β-1, 4-xylanase in *S. sclerotiorum*. The *SsXyl1* expression showed a slight increase during the development and germination stages of sclerotia and a dramatic increase during infection. The expression of *SsXyl1* was induced by xylan. The *SsXyl1* deletion strains produce aberrant sclerotia that could not germinate to form apothecia. The *SsXyl1* deletion strains also lost virulence to the hosts. This study demonstrates the important roles of endo-β-1, 4-xylanase in the growth and virulence of *S. sclerotiorum.*

## Introduction

*Sclerotinia sclerotiorum* (Lib.) de Bary is a ubiquitous necrotrophic fungal pathogen that infects more than 400 plant species worldwide resulting in significant losses in many cultivated crops such as oilseed, sunflower, soybean, and common bean (Purdy, 1979; Boland and Hall, 1994; Bolton et al., 2006).

*Sclerotinia sclerotiorum* produces sclerotia—the primary long-term structures composed of compact vegetative hyphal cells. In a suitable environment, the sclerotia can germinate to form apothecia, which release a huge number of ascospores that cause crop sclerotioses (Bolton et al., 2006). The carpogenic germination of sclerotia is a key process underlying diseases caused by *S. sclerotiorum.* It is highly significant in an economical context. Sclerotial germination is a complex biological process that is controlled by both external environmental conditions and internal natural factors. Many factors have been shown to play important roles in sclerotial germination including temperature (Clarkson et al., 2003, 2007), soil humidity, ventilation condition and the depth of burial (Wu and Subbarao, 2008).

Some cellular signal transduction pathways have been shown to regulate sclerotial germination. Disruption of adenylate cyclase gene *Sac1* in *S. sclerotiorum* decreases cAMP and impairs carpogenic germination of sclerotia suggesting that the cAMP signal pathway regulates the sclerotial germination (Jurick and Rollins, 2007). The process was also influenced by glutathione accumulation because the sclerotia produced by the strain that lost the gamma-glutamyl transpeptidase gene *Ss-Ggt1* failed to produce apothecia (Li et al., 2012). Furthermore, some secreted proteins located at the cell wall were identified as being associated with the sclerotial germination of *S. sclerotiorum* (Zhu et al., 2013; Xiao et al., 2014). However, analysis of the molecular mechanisms underlying the carpogenic germination of sclerotia is still in its infancy.

As a necrotrophic fungus, *S. sclerotiorum* secretes many cell wall-degrading enzymes (CWDEs) to degrade the plant cell wall components and feed on the tissues (Lumsden, 1969; Riou et al., 1991; Alghisi and Favaron, 1995). The fungus produces several forms of pectinases such as polygalacturonases (PGs) to degrade pectin—the major constituent of the plant cell wall (Waksman et al., 1991; Reymond et al., 1994; Cotton et al., 2003; Favaron et al., 2004; Li et al., 2004; Kasza et al., 2004). PGs genes encode important virulence factors in many important fungal pathogens indicating a universal role in the pathogenicity in *S. sclerotiorum* (Wagner et al., 2000; García-Maceira et al., 2001; Kars et al., 2005). In addition to the PGs, Yajima et al. (2009) reported that an arabinofuranosidase/β-xylosidase precursor gene disruption mutant of *S. sclerotiorum* showed reduced virulence on canola tissue.

Xylan is the major constituent of hemicelluloses in plant cell walls. It consists principally of xylose and arabinose (Bastawde, 1992). The main chain of xylan is composed of β-(1–4) linked β-xylopyranose. The degradation of xylan requires the incorporation of several hydrolytic enzymes. Of those, endo-β-1, 4-xylanase (EC 3.2.1.8) is the most crucial enzyme (Collins et al., 2005). Xylanases are mainly divided into two families of glycosyl hydrolases: family F or GH10 and family G or GH11 based on amino acid sequences (Jeffries, 1996; Zhou et al., 2008). Due to the important roles of xylanases in the infection processes in pathogens, many genes in fungal pathogens that encode xylanases have been identified and characterized. Most xylanase genes are not essential for pathogenicity (Apel et al., 1993; Gómez-Gómez et al., 2001; Wu et al., 2006; Sella et al., 2013). However, Brito et al. (2006) reported that an endo-β-1, 4-xylanase encoding gene *xyn11A* is required for virulence in *Botrytis cinerea*. Until now, functional characterization of the xylanase gene in *S. sclerotiorum* at the molecular biology level remains rare.

In this research, a gene named *SsXyl1* (Accession No. XM_001591074, SS1G_07749) was predicted to encode an endo-β-1, 4-xylanase in *S. sclerotiorum*. The gene was characterized with reverse-genetic methods, and its function in pathogenicity and development was analyzed. The results will assist in developing our understanding of the mechanism underlying its pathogenicity and development of *S. sclerotiorum*.

## Materials and Methods

### Fungal Strains and Culture Conditions

In this study, *S. sclerotiorum* isolate 1980 (Godoy et al., 1990) was used as the wild-type strain. Strains were routinely cultured at 20°C on potato dextrose agar (PDA; Difco Laboratories, Detroit, MI, USA). Gene deletion transformants were cultured on PDA with hygromycin B at 100 μg/ml (Calbiochem, San Diego, CA, USA). Gene complemented strains were cultured on PDA with hygromycin B at 100 μg/ml and G-418 at 30 μg/ml (Sigma, St. Louis, MO, USA).

### Sequence Analysis and Alignment

*SsXyl1* gene was obtained through the BLAST searches for homologous sequences in *S. sclerotiorum* genome using reported xylanases gene in *B. cinere* (Brito et al., 2006) and some other plant pathogenic fungi. The signal peptide sequence and conserved domain were predicted using the SignalP 4.1 Server^1^ and PFAM^2^, respectively. The sequence alignments were carried out using DNAMAN software (Lynnon BioSoft, Vaudreuil, QC, Canada) and displayed with GeneDoc software (Nicholas and Nicholas, 1997). Conserved amino acids were shown with a shaded background. The phylogenetic tree was constructed using maximum likelihood analysis in MEGA (Tamura et al., 2011).

### Xylanase Activity Assays

To assay the xylanase activity of SsXyl1, the cDNA fragment encoding the amino acid T^22^ to S^222^ of SsXyl1 (without signal peptide) was artificially synthesized (Shengong, Shanghai, China) and inserted into pPICZαA. The resulting vector was transformed into *Pichia pastoris* X33 strain via electroporation. The strain was cultured in BMMY medium (1% yeast extract, 2% peptone, 100 mM potassium phosphate (pH 6.0), 1.34% yeast nitrogen base with ammonium sulfate, 0.00004% biotin, and 0.5% methanol) at 28°C under shaking (220 rpm) for 48 h. The culture filtrate was collected and the xylanase activity was measured via the 3, 5-dinitrosalicylic acid (DNS) method (Miller, 1959; Karaoglan et al., 2014). To measure the xylanse activity, 900 μl 1.0% beechwood xylan (Sigma, St. Louis, MO, USA) in 50 mM sodium citrate buffer (pH 5.0) were incubated 5 min at 50°C. One hundred microliters of samples (culture filtrate) were added in the xylan solution followed by incubation 5 min at 50°C. Next, 9 ml DNS solution was added to the reaction mixture. The mixture was then boiled for 5 min, and the absorbance was measured at 540 nm. Xylose (0–6 μmol) was used to create a standard curve. One unit of xylanase activity was defined as the amount of enzyme catalyzing the formation of 1.0 μmol of xylose per minute at pH 5.0 at 50°C. The culture filtrate of the strain transformed with pPICZαA as the control.

### RT-PCR and Real-Time RT-PCR

A reverse-transcriptase polymerase chain reaction (RT-PCR) was applied to determine the relative expression levels of *SsXyl1* during the different development stages and the infection processes of the *S. sclerotiorum*. The wild-type strains were inoculated on the PDA medium and *Arabidopsis thaliana* as described by Yu et al. (2015) followed by mycelia harvest. To compare the expression level of *SsXly1* on different carbon sources, the wild-type strains were cultured on different minimum medium in which the glucose was replaced with xylan or tomato leaf extract. The total RNA in each sample was extracted with Trizol (Huashun, China). The first-strand of cDNA synthesis was performed with RevertAid First Strand cDNA Synthesis Kit (Thermo Scientific, Waltham, MA, USA). The real-time RT-PCR was applied on a Bio-Rad CFX96^TM^ Realtime System (Hercules, CA, USA). The *SsXyl1* cDNA abundance was normalized using the β*-tubulin* gene (*tub1*, SS1G_04652) as an internal control. The primer pairs qRT-Xyl1fp (TTCTCGGCAATTTCTACATCC)/qRT-Xyl1rp (CCCAATCTACAGTGAAGGAGC) and qRT-Tubfp (GTGAGGCTGAGGGCTGTGA)/qRT-Tubrp (CCTTTGGCGATGGGACG) were designed with Primer Premier 6.0 (Premier, Canada) and used to amplify the cDNA of *SsXyl1* and *tub1*. The amplification mixtures were composed of 10 μl of SYBR Green Realtime PCR Master Mix (Toyobo, Japan), with 4 pM primer, 1 μl of cDNA, and ddH_2_O water to a final volume of 20 μl. Amplification steps were as follows: 95°C for 2 min (1 cycle) followed by 95°C for 20 s, 58°C for 15 s and 72°C for 20 s (40 cycles). Each sample was analyzed over three biological replicates and each real-time RT-PCR analysis was repeated three times.

The RT-PCR was used to analyze *SsXyl1* expressions in the gene deletion and complemented strains. The transforms were cultured on PDA supplemented with different antibiotics for 4 days, and the mycelia were harvested. The total RNA extraction and cDNA synthesis were performed as mentioned before. The primer pairs RT-Xyl1fp (CTTCACTGTAGATTGGGACACC)/RT-Xyl1rp (ACTAACCGTTTGCGTAGCAG) were amplified a 460-bp fragment of *SsXyl1* cDNA. The *tub* expressions amplified with qRT-Tubfp/qRT-Tubrp served as the control.

### Generation of Gene Deletion and Complementation Strains

The gene deletion vector was constructed based on vector pSKH (Hamid et al., 2013). A primer pair Xyl1DF (CGCAAGCTTGGGGAGAGAGATATGCAAATGT) and Xyl1DR (CCGCTCGAGAGAGGCTTTTAGTTTCACAATG) were designed to amplify a 1000-bp of 3′ untranslated region (UTR) of *SsXyl1* gene. The fragment was digested with *Hind*III and *Xho*I and then inserted into pSKH to produce the pSKHXYL1. The primer pairs Xyl1UF (GCCGAGCTCATGGAAATAAACACGCCTAGC) and Xyl1UR (GCCGTCGACCCACGCAGTGCTTTATATACT) were amplified a 1000-bp of 5′ UTR of *SsXyl1* gene. The fragment was then digested with *Sac*I and *Sal*I and inserted into pSKHXYL1 to generate the pSKHXYL2. The construct was used as a PCR template to generate split-marker fragments. The 5′UTR::N-HY was amplified using primers Xyl1U (CATGGAAATAAACACGCCTAGC) and HY (AAATTGCCGTCAACCAAGCTC). The 3′UTR::C-YG was generated using YG (TTTCAGCTTCGATGTAGGAGG) and Xyl1D (GAGAGGCTTTTAGTTTCACAATG). The overlap between the two hygromycin resistance gene (*hph*) fragments was 741 bp. The two fragments were used to transform into *S. sclerotiorum* wild-type protoplasts as described by Rollins (2003).

To construct the gene complement vector, a neomycin-resistance gene was inserted into pCAMBIA3300 at the *Xba*I site to produce pCAMBIA3300NEO. A pair of primers 07749FP (CCGGAATTCTTCTATGCACGCAGACTTATTG) and 07749RP (CCGCTCGAGGTCAAATACAACCTGCCACCT) were designed to amplify the genome DNA from the wild-type strain and yield 2.8-kb fragments that contained the *SsXyl1* coding sequence and 1 kb of 5′ and 1 kb of 3′ untranslated region. The fragment was digested with *Eco*RI and *Xho*I and then inserted into pCAMBIA3300NEO to produce the pCXYL1. The vector was then linearized with *Xho*I and transformed into the KO52 protoplasts with PEG methods (Rollins, 2003).

### Pathogenicity Assays

The pathogenicity assays were performed on leaves of *Brassica napus* Zhongshuang 9 and *A. thaliana* Columbia-0. *B. napus* were grown in a greenhouse at 25°C to 35°C for about 10 weeks. *A. thaliana* were cultivated in a growth chamber at 25°C with a 12 h light/12 h dark cycle for 5 weeks. The leaves of *B. napus* and *A. thaliana* were inoculated with mycelia-colonized agars (φ = 6 mm) obtained from actively growing colony margins of the wild-type, gene deletion, and complemented strains. The inoculated plants were maintained at 90% relative humidity. Photographs were taken at 72 h post inoculation (hpi) for rapeseed leaves and at 120 hpi for *A. thaliana*. The experiment was repeated three times and five plants were inoculated with each strain for each repeat.

## Results

### SsXyl1 Characterization

**FIGURE 1:**
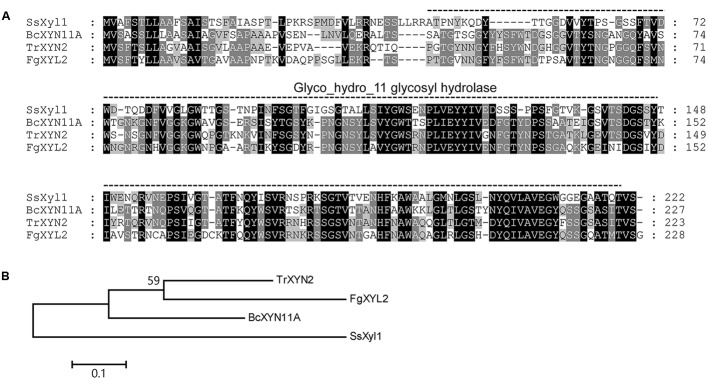
**Multiple alignment of the amino acid sequences of SsXyl1 with the sequences of *B. cinerea* XYN11A, *Trichoderma reesei* XYN2 and *Fusarium graminearum* XYL2. (A)** Multiple sequence alignment with identical amino acid residues shaded. The predicated Glyco_hydro_11 glycosyl hydrolase motify is indicated. Numbers correspond to the amino acid of the proteins. **(B)** Phylogenetic tree constructed based on the amino acid multiple sequence alignment of SsXyl homologous.

The *SsXyl1* gene contains an ORF of 669 bp and encodes a 222-amino-acid polypeptide. The N-terminal of SsXyl1 was predicted to contain a typical peptide with SignalP 4.1 Server (Petersen et al., 2011). The predicted cleavage site was between amino acid position 21 and 22. This generates a putative mature protein with a calculated molecular mass of 21.69 kDa and an isoelectric point (pI) of 4.82. Residues 46–220 in the protein were predicted by PFAM (Finn et al., 2015) to be a Glyco_hydro_11 glycosyl hydrolase motif. Sequence comparison showed that SsXyl1 exhibited high sequence similarity with endo-β-1, 4-xylanase of *B. cinerea* XYN11A (53% identities, *E*-value: 5*e*-50) (Brito et al., 2006), *Trichoderma reesei* XYN2 (45% identities, *E*-value: 2*e*-55) (Enkerli et al., 1999) and *Fusarium graminearum* XYL2 (41% identities, *E*-value: 3*e*-46) (Dong et al., 2012). The sequence alignment and phylogenetic tree are shown in **Figure 1**.

To confirm the xylanase activity of SsXyl1, the *SsXyl1* cDNA (without signal peptide) was linked into pZICZαA. The resulting vector was transformed into the yeast *P. pastoris* X33 strain. The strains were cultured with shaking, and the xylanase activity of culture filtrate was determined. The results showed that the enzyme activity for the strain carrying *SsXyl1* was 39.17 U/ml, and the wild-type strain was 0.47 U/ml. These results indicate that *SsXyl1* encodes a xylanase in *S. sclerotiorum*.

### *SsXyl1* Expression Patterns

**FIGURE 2:**
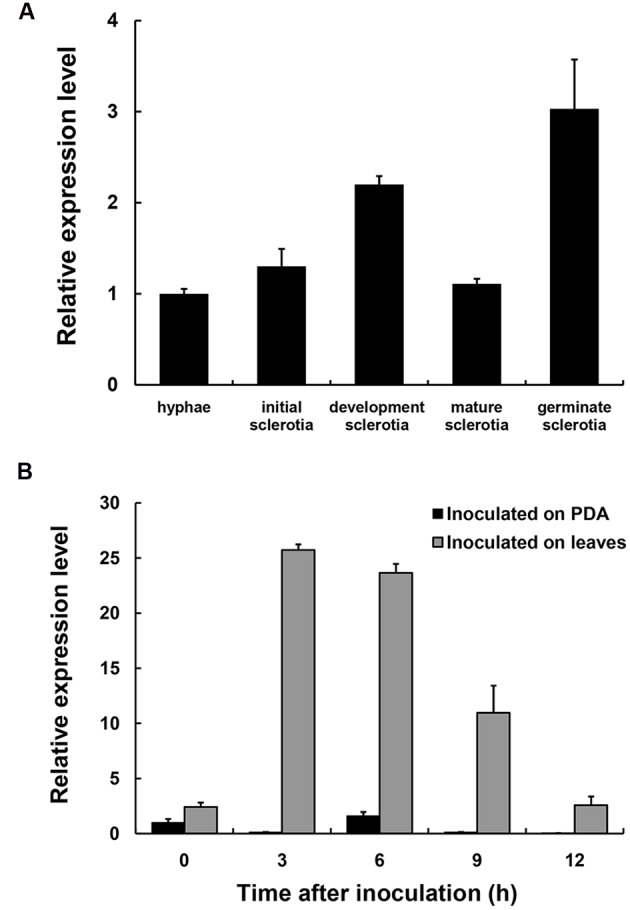
**Expression analysis of *SsXly1* during various stages. (A)** Real-time reverse-transcriptase polymerase chain reaction (RT-PCR) analysis of transcript accumulation during different development stages of the sclerotia and germinated sclerotia. **(B)** Transcript accumulation analysis during infection. The quantity of the *SsXly1* cDNA in each sample was normalized to that of *tub1* cDNA. The relative abundance of *SsXly1* cDNA in the stage of hyphae growth or in mycelium at 0 h was normalized to one. Bars indicate the standard error. The analyses were repeated three times. One replicate is shown.

The expression levels of *SsXyl1* during the different development stages and the infection process were determined with real-time RT-PCR. **Figure 2A** shows that the *SsXyl1* expression was slightly increased during the development and carpogenic germination stages of sclerotia. When inoculated on the *A. thaliana*, the *SsXly1* expression was dramatically increased at 3 hpi and then decreased gradually. The expression was still higher than at 0 dpi (**Figure 2B**). The results suggest that *SsXyl1* expression is strongly induced during infection.

**FIGURE 3:**
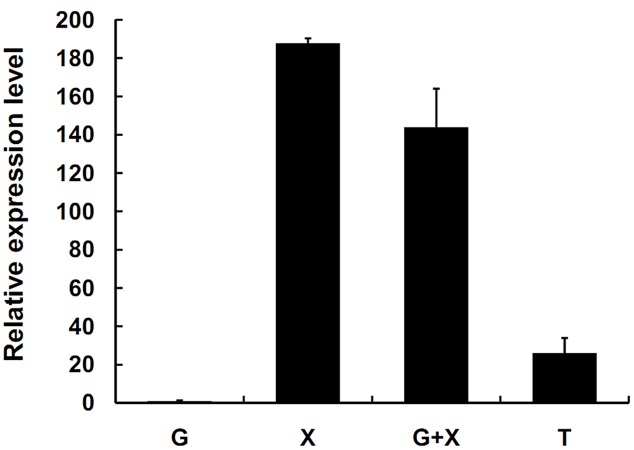
**Expression analysis of *SsXyl1* on different carbon sources.** The level of *SsXly1* mRNA in each sample was normalized to the levels of *tub1* mRNA. The strains were cultured in minimal media with glucose (G), xylan (X), glucose+xylan (G+X), and tomato extracts (T) as carbon sources. The relative level of *SsXly1* mRNA in mycelium cultured in medium with glucose as carbon sources was normalized to one. Bars indicate the standard error. The analyses were repeated three times. One replicate is shown.

*SsXyl1* expression was also determined in the mycelia of *S. sclerotiorum* grown on different carbon sources. **Figure 3** shows that the expression level of *SsXyl1* for the mycelia grown in xylan was significantly higher than that grown in glucose. The level was maximal when xylan was the only carbon source. The expression pattern indicates that *SsXyl1* expression was induced by xylan. The *SsXly1* expression is also induced when the tomato leaf extracts were the carbon source.

### Role of *SsXyl1* in Fungal Growth and Development

To functional analysis the *SsXly1* in *S. sclerotiorum*, *SsXyl1* gene deletion vector was constructed based on the vector pSKH as described in the Section “Materials and Methods.” The construct was used as the PCR template to generate split-marker fragments, which transformed the protoplasts of the wild-type strain (**Supplementary Figure S1A**). Several transformants were obtained and confirmed by amplifying of the hygromycin resistance gene. Some strains were chosen randomly and the *SsXyl1* expressions were determined with RT-PCR. The results showed that KO52 strains lacked the *SsXyl1* transcript (**Supplementary Figure S1B**). The complementation strain KOC5 was generated via the transformation of the *SsXyl1* complemented vector into the KO52 protoplasts. RT-PCR revealed that the *SsXyl1* transcript was present in KOC5 (**Supplementary Figure S1B**).

**FIGURE 4:**
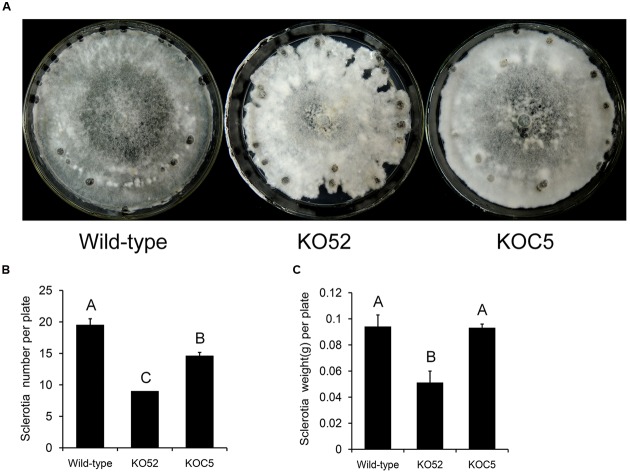
**Deletion of *SsXyl1* has significant effects on colony morphology and sclerotial development. (A)** Phenotype of wild-type strain, *SsXyl1* deletion (KO52) and complemented (KOC5) strains on potato dextrose agar (PDA) plates for 15 days. **(B)** The number of sclerotial produced in 9 cm per petri plates. **(C)** Sclerotia mass per plate. Different letters on a graph indicate significant differences, *P* < 0.05.

**FIGURE 5:**
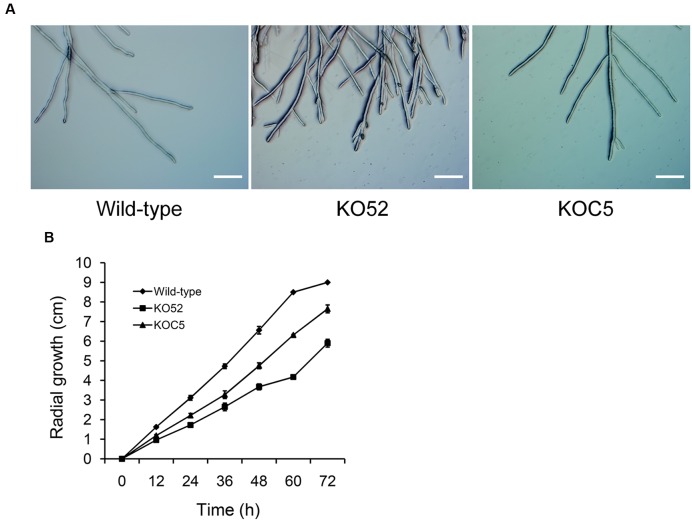
**Deletion of *SsXyl1* has significant effects on hyphal growth. (A)** Hyphal morphology of wild-type, *SsXyl1* deletion (KO52) and complemented (KOC5) strains. **(B)** Radial growth of wild-type, KO52 and KOC5. The strains were cultured on PDA plates and the colony diameters were measured every 12 h. Bar = 200 μm.

**Figure 4A** shows the colonial morphology of the wild-type, *SsXyl1* deletion (KO52), and complemented (KOC5) strains on PDA plates. The gene deletion strain showed abnormal morphology versus the wild-type strain. The strain produced fewer sclerotia than the wild-type strain. **Figures 4B,C** indicates that the number and weight of sclerotia for KO52 were approximately half of those of the wild-type strain. Most importantly, sclerotia produced by the KO52 cannot produce apothecia under standard conditions of carpogenic germination. The KO52 exhibited a dense hyphal branch when observed by microscopy (**Figure 5A**). It has a slower growth rate versus the wild-type strain (**Figure 5B**).

### Role of *SsXyl1* in Virulence

**FIGURE 6:**
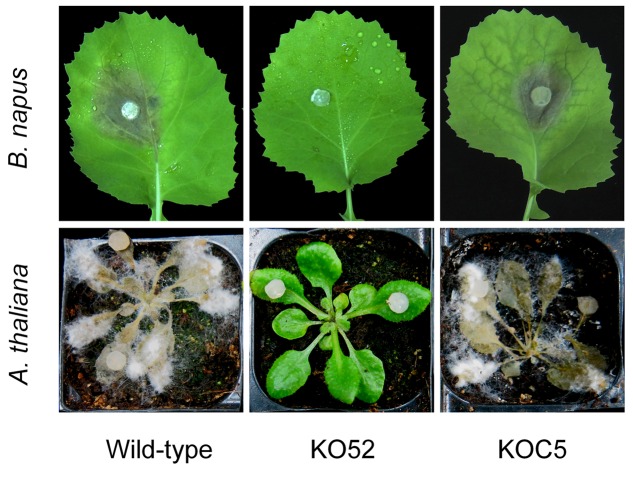
**Virulence analysis on leaves of rapeseed (A)** and *A. thaliana* **(B)**, following inoculation of the wild-type strain, *SsXyl1* deletion (KO52) and complemented (KOC5) strains. The experiment was repeated three times, and each strain was performed with five rapeseed leaves or *A. thaliana* plants.

The wild-type, *SsXyl1* deletion, and complemented strains were all inoculated on the detached leaves of oilseed rape and *A. thaliana* plants. **Figure 6** shows that the gene deletion mutant KO52 lost the virulence to the hosts, while the re-induction of *SsXyl1* in the deletion mutant almost completely rescued the phenotype. The results suggest that *SsXly1* plays an important role in the virulence of *S. sclerotiorum*.

## Discussion

In this report, we described an endo-β-1, 4-xylanase gene named *SsXyl1* in *S. sclerotiorum.* SsXyl1 was predicted to contain a GH11 glycosyl hydrolase motif with xylanase activities. *SsXyl1* was up-regulated during the development and carpogenic germination of sclerotia. The *SsXyl1* deletion strains produced aberrant sclerotia that were defective in carpogenic germination. *SsXyl1* was also significantly up-regulated during the infection processes; gene deletion strains lost virulence to the hosts. Our results suggest that *SsXyl1* plays an essential role in the growth and virulence of *S. sclerotiorum*.

SsXyl1 is believed to belong to the GH11 group because of the GH11 glycosyl hydrolase motif, its low molecular weight, and the basic pI. The GH11 group was the best characterized GH group with bacterial and fungal members considered to be a true xylanase because of their high substrate specificity (Paës et al., 2012). Here, the xylanase activity of SsXyl1 was confirmed using the 3, 5-DNS method. Fungal plant pathogens colonize their hosts via the production of different CWDEs including pectinases, cellulases, xylanses, and cutinases (Annis and Goodwin, 1997; ten Have et al., 2002; Wanjiru et al., 2002; D’Ovidio et al., 2004; Reignault et al., 2008). In *S. sclerotiorum*, enzymes such as petinase, cellulose, proteases, and glucoamylases can facilitate the invasion and colonization of host tissue (Riou et al., 1991; Martel et al., 1998, 2002; Poussereau et al., 2001a,b). Our results indicate a role of xylanses in the pathogenicity of this fungus.

Until now, many genes that encode xylanases in fungal pathogens have been cloned and functionally analyzed, but most have no relationship with the fungal pathogenicity (Wu et al., 1997; Gómez-Gómez et al., 2002). In this research, *SsXyl1* shows a high level of expression during the infection and gene deletion strains lost virulence to many hosts. These findings indicate that the gene is essential for the virulence of *S. sclerotiorum*. In *B. cinerea*, *xyn11A* encodes an endo-β-1, 4-xylanase Xyn11A and deletion of the gene has a more pronounced effect on virulence (Brito et al., 2006). Xyn11A contributes to virulence with its necrotizing activity not with its catalytic activity (Noda et al., 2010). The similarity between the xylanases SsXyl1 and Xyn11A in the amino acid (53% identity, 67% positive; **Figure 1**) seems to indicate that SsXyl1 also contributes to the virulence via the necrotizing activity, but more evidence is needed.

Carpogenic germination of sclerotia is a key process in Sclerotinia diseases. Until now, many genes associated with cell signal transduction pathway have been confirmed to control this process in *S. sclerotiorum* (Jurick and Rollins, 2007; Li et al., 2012), but the proteins that worked downstream of the signal transduction pathway to control sclerotial germination have remained largely unknown. In this research, the sclerotia for *SsXyl1* deletion mutants cannot germinate to form apothecia suggesting that *SsXyl1* is related to the carpogenic germination. To the best of our knowledge, this is the first report to show that the xylanase is related to the sclerotial germination in fungi.

The carpogenic germination of sclerotia can be divided into three stages: (1) carpophores initials form in inner medullary tissues of sclerotia; (2) the carpophores arise through the ruptured rind; and (3) the tips of the carpophores become modified to form apothecia (Coley-Smith and Cooke, 1971). Once a sclerotial rind is broken or damaged, the sclerotia germinate more rapidly (Makkonen and Pohjakallio, 1960). As the resting structure of *S. sclerotiorum*, the sclerotia rind forms a thick cell wall that could maintain internal water and energy. The thick cell wall contains the primary cell wall, cellulose, hemicelluloses as well as some yet-to-be-identified materials surrounding it (Willetts and Bullock, 1992; Young and Ashford, 1992). Thus, the sclerotial rind plays an important role in the inhibition of sclerotial germination (Garg et al., 2010). We hypothesized that the sclerotia use enzymes to hydrolyze the thick cell wall and degrade it before germinating. SsXyl1 may be an important candidate enzyme, but the hydrolytic activity of SsXyl1 to the cell wall of the sclerotia requires more study.

## Author Contributions

Conceived and designed the experiments: YY, JX, JD, YHY, CB, and LQ. Performed the experiments: YY and JX. Analyzed the data: YY and JX. Contributed reagents/materials/analysis tools: JD, YHY, CB, and LQ. Wrote the paper: YY and JX. All authors read and approved the final manuscript.

## Conflict of Interest Statement

The authors declare that the research was conducted in the absence of any commercial or financial relationships that could be construed as a potential conflict of interest.

## Footnotes

1 http://www.cbs.dtu.dk/services/SignalP/

2 http://pfam.xfam.org/

## Supplementary material

FIGURE S1 **Disruption of the *SsXyl1* gene function using a split marker strategy. (A)** Schematic describing an overlapping hygromycin phosphotransferase gene (*hph*) marker for *SsXyl1* disruption. The N-HY and C-YG imply 741 bp overlapping the 5′ and 3′fragments of *hph* gene, respectively. **(B)** RT-PCR analysis the expressions of *SsXyl1* in the wild-type, gene deletion (KO52), and complemented (KOC5) strains. The expression of the *Tub* gene was the internal control.

## References

[B1] Alghisi P. Favaron F. (1995). Pectin-degrading enzymes and plant-parasite interactions. *Eur. J. Plant Pathol.* 101 365–375. 10.1007/BF01874850

[B2] Annis S. L. Goodwin P. H. (1997). Recent advances in the molecular genetics of plant cell wall-degrading enzymes produced by plant pathogenic fungi. *Eur. J. Plant Pathol.* 103 1–14. 10.1023/A:1008656013255

[B3] Apel P. A. Panaccione D. G. Holden F. R. Walton J. D. (1993). Cloning and targeted gene disruption of XYL1, a 1, 4-xylanase gene from the maize pathogen *Cochliobolus carbonum*. *Mol. Plant Microbe Interact.* 6 467–467. 10.1094/MPMI-6-467 8400376

[B4] Bastawde K. B. (1992). Xylan structure, microbial xylanases, and their mode of action. *World J. Microbiol. Biotechnol.* 8 353–368. 10.1007/BF01198746 24425504

[B5] Boland G. J. Hall R. (1994). Index of plant hosts of *Sclerotinia sclerotiorum*. *Can. J. Plant Pathol.* 16 93–108. 10.1080/07060669409500766

[B6] Bolton M. D. Thomma B. P. H. J. Nelson B. D. (2006). *Sclerotinia sclerotiorum* (Lib.) de Bary: biology and molecular traits of a cosmopolitan pathogen. *Mol. Plant Pathol.* 7 1–16. 10.1111/j.1364-3703.2005.00316.x 20507424

[B7] Brito N. Espino J. J. González C. (2006). The endo-β-1, 4-xylanase Xyn11A is required for virulence in *Botrytis cinerea*. *Mol. Plant Microbe Interact.* 19 25–32. 10.1094/MPMI-19-0025 16404950

[B8] Clarkson J. P. Phelps K. Whipps J. M. Young C. S. Smith J. A. Watlting M. (2007). Forecasting *Sclerotinia* disease on lettuce: a predictive model for carpogenic germination of sclerotia. *Phytopathology* 97 621–631. 10.1094/PHYTO-97-5-0621 18943582

[B9] Clarkson J. P. Staveley J. Phelps K. Young C. S. Whipps J. M. (2003). Ascospore release and survival in *Sclerotinia sclerotiorum*. *Mycol. Res.* 107 213–222. 10.1017/S0953756203007159 12747333

[B10] Coley-Smith J. R. Cooke R. C. (1971). Survival and germination of fungal sclerotia. *Annu. Rev. Phytopathol.* 9 65–92. 10.1146/annurev.py.09.090171.000433

[B11] Collins T. Gerday C. Feller G. (2005). Xylanases, xylanase families and extremophilic xylanases. *FEMS Microbiol. Rev.* 29 3–23. 10.1016/j.femsre.2004.06.005 15652973

[B12] Cotton P. Kasza Z. Bruel C. Rascle C. Fèvre M. (2003). Ambient pH controls the expression of endopolygalacturonase genes in the necrotrophic fungus *Sclerotinia sclerotiorum*. *FEMS Microbiol. Lett.* 227 163–169. 10.1016/S0378-1097(03)00582-2 14592704

[B13] Dong X. Meinhardt S. W. Schwarz P. B. (2012). Isolation and characterization of two endoxylanases from *Fusarium graminearum*. *J. Agric. Food Chem.* 60 2538–2545. 10.1021/jf203407p 22313372

[B14] D’Ovidio R. Mattei B. Roberti S. Bellincampi D. (2004). Polygalacturonases, polygalacturonase-inhibiting proteins and pectic oligomers in plant-pathogen interactions. *Biochim. Biophys. Acta* 1696 237–244. 10.1016/j.bbapap.2003.08.012 14871664

[B15] Enkerli J. Felix G. Boller T. (1999). The enzymatic activity of fungal xylanase is not necessary for its elicitor activity. *Plant Physiol.* 121 391–398. 10.1104/pp.121.2.391 10517830 PMC59401

[B16] Favaron F. Sella L. D’Ovidio R. (2004). Relationships among endo-polygalacturonase, oxalate, pH, and plant polygalacturonase-inhibiting protein (PGIP) in the interaction between *Sclerotinia sclerotiorum* and soybean. *Mol. Plant Microbe Interact.* 17 1402–1409. 10.1094/MPMI.2004.17.12.1402 15597746

[B17] Finn R. D. Coggill P. Eberhardt R. Y. Eddy S. R. Mistry J. Mitchell A. L. (2015). The Pfam protein families database: towards a more sustainable future. *Nucl. Acids Res.* 44 279–285. 10.1093/nar/gkv1344 PMC4702930 26673716

[B18] García-Maceira F. I. Di Pietro A. Huertas-González M. D. Ruiz-Roldán M. C. Roncero M. I. (2001). Molecular characterization of an endopolygalacturonase from *Fusarium oxysporum* expressed during early stages of infection. *Appl. Environ. Microbiol.* 67 2191–2196. 10.1128/AEM.67.5.2191-2196.2001 11319099 PMC92854

[B19] Garg H. Sivasithamparam K. Barbetti M. J. (2010). Scarification and environmental factors that enhance carpogenic germination of sclerotia of *Sclerotinia sclerotiorum*. *Plant Dis.* 94 1041–1047. 10.1094/PDIS-94-8-1041 30743489

[B20] Godoy G. Steadman J. R. Dickman M. B. Dam R. (1990). Use of mutants to demonstrate the role of oxalic acid in pathogenicity of *Sclerotinia sclerotiorum* on *Phaseolus vulgaris*. *Physiol. Mol. Plant Pathol.* 37 179–191. 10.1016/0885-5765(90)90010-U

[B21] Gómez-Gómez E. Roncero I. M. Di Pietro A. Hera C. (2001). Molecular characterization of a novel endo-β-1, 4-xylanase gene from the vascular wilt fungus *Fusarium oxysporum*. *Curr. Genet.* 40 268–275. 10.1007/s00294-001-0260-0 11795847

[B22] Gómez-Gómez E. Ruíz-Roldán M. C. Di Pietro A. Roncero M. I. G. Hera C. (2002). Role in pathogenesis of two endo-β-1, 4-xylanase genes from the vascular wilt fungus *Fusarium oxysporum*. *Fungal Genet. Biol.* 35 213–222. 10.1006/fgbi.2001.1318 11929211

[B23] Hamid M. I. Zeng F. Cheng J. Jiang D. Fu Y. (2013). Disruption of heat shock factor 1 reduces the formation of conidia and thermotolerance in the mycoparasitic fungus *Coniothyrium minitans*. *Fung. Genet. Biol.* 53 42–49. 10.1016/j.fgb.2012.12.002 23357354

[B24] Jeffries T. W. (1996). Biochemistry and genetics of microbial xylanases. *Curr. Opin. Biotech.* 7 337–342. 10.1016/S0958-1669(96)80041-3 8785441

[B25] Jurick W. M. Rollins J. A. (2007). Deletion of the adenylate cyclase (*sac1*) gene affects multiple developmental pathways and pathogenicity in *Sclerotinia sclerotiorum*. *Fung. Genet. Biol.* 44 521–530. 10.1016/j.fgb.2006.11.005 17178247

[B26] Karaoglan M. Yildiz H. Inan M. (2014). Screening of signal sequences for extracellular production of *Aspergillus niger* xylanase in *Pichia pastoris*. *Biochem. Eng. J.* 92 16–21. 10.1016/j.bej.2014.07.005

[B27] Kars I. Krooshof G. H. Wagemakers L. Joosten R. Benen J. A. van Kan J. A. (2005). Necrotizing activity of five *Botrytis cinerea* endopolygalacturonases produced in *Pichia pastoris*. *Plant J.* 43 213–225. 10.1111/j.1365-313X.2005.02436.x 15998308

[B28] Kasza Z. Vagvölgyi C. Févre M. Cotton P. (2004). Molecular characterization and in planta detection of *Sclerotinia sclerotiorum* endopolygalacturonase genes. *Curr. Microbiol.* 48 208–213. 10.1007/s00284-003-4166-6 15057467

[B29] Li M. Liang X. Rollins J. A. (2012). *Sclerotinia sclerotiorum* γ-glutamyl transpeptidase (Ss-Ggt1) is required for regulating glutathione accumulation and development of sclerotia and compound appressoria. *Mol. Plant Microbe Interact.* 25 412–420. 10.1094/MPMI-06-11-0159 22046959

[B30] Li R. Rimmer R. Buchwaldt L. Sharpe A. G. Séguin-Swartz G. Hegedus D. D. (2004). Interaction of *Sclerotinia sclerotiorum* with *Brassica napus*: cloning and characterization of endo- and exo-polygalacturonases expressed during saprophytic and parasitic modes. *Fungal Genet. Biol.* 41 754–765. 10.1016/j.fgb.2004.03.002 15219560

[B31] Lumsden R. D. (1969). *Sclerotinia sclerotiorum* infection of bean and the production of cellulase. *Phytopathology* 59 653–657.

[B32] Makkonen R. Pohjakallio O. (1960). On the parasites attacking the sclerotia of some fungi pathogenic to higher plants and on the resistance of these sclerotia to their parasites. *Acta Agric. Scand.* 10 105–126. 10.1080/00015126009434141

[B33] Martel M. B. Hervé du Penhoat C. Létoublon R. Fèvre M. (2002). Purification and characterization of a glucoamylase secreted by the plant pathogen *Sclerotinia sclerotiorum*. *Can. J. Microbiol.* 48 212–218. 10.1139/w02-011 11989765

[B34] Martel M. B. Létoublon R. Fèvre M. (1998). Purification and characterization of two endopolygalacturonases secreted during the early stages of the saprophytic growth of *Sclerotinia sclerotiorum*. *FEMS Microbiol. Lett.* 158 133–138. 10.1111/j.1574-6968.1998.tb12812.x 9453165

[B35] Miller G. L. (1959). Use of dinitrosalicylic acid reagent for determination of reducing sugar. *Anal. Chem.* 31 426–428. 10.1021/ac60147a030

[B36] Nicholas K. B. Nicholas H. B. J. (1997). *GeneDoc: A Tool for Editing and Annotating Multiple Sequence Alignments*. Available at: http://www.citeulike.org/user/gwallau/article/6113940

[B37] Noda J. Brito N. González C. (2010). The *Botrytis cinerea* xylanase Xyn11A contributes to virulence with its necrotizing activity, not with its catalytic activity. *BMC Plant Biol.* 10:38 10.1186/1471-2229-10-38 PMC2844071 20184750

[B38] Paës G. Berrin J. G. Beaugrand J. (2012). GH11 xylanases: structure/function/properties relationships and applications. *Biotechnol. Adv.* 30 564–592. 10.1016/j.biotechadv.2011.10.003 22067746

[B39] Petersen T. N. Brunak S. von Heijne G. Nielsen H. (2011). SignalP 4.0: discriminating signal peptides from transmembrane regions. *Nat. Methods* 8 785–786. 10.1038/nmeth.1701 21959131

[B40] Poussereau N. Creton S. Billon-Grand G. Rascle C. Fevre M. (2001a). Regulation of acp1, encoding a non-aspartyl acid protease expressed during pathogenesis of *Sclerotinia sclerotiorum*. *Microbiology* 147 717–726. 10.1099/00221287-147-3-717 11238979

[B41] Poussereau N. Gente S. Rascle C. Billon-Grand G. Fèvre M. (2001b). *aspS* encoding an unusual aspartyl protease from *Sclerotinia sclerotiorum* is expressed during phytopathogenesis. *FEMS Microbiol. Lett.* 194 27–32. 10.1111/j.1574-6968.2001.tb09441.x 11150661

[B42] Purdy L. H. (1979). *Sclerotinia sclerotiorum*: history, diseases and symptomatology, host range, geographic distribution, and impact. *Phytopathology* 69 875–880. 10.1094/Phyto-69-875

[B43] Reignault P. Valette-Collet O. Boccara M. (2008). The importance of fungal pectinolytic enzymes in plant invasion, host adaptability and symptom type. *Eur. J. Plant pathol.* 120 1–11. 10.1007/s10658-007-9184-y

[B44] Reymond P. Deléage G. Rascle C. Fèvre M. (1994). Cloning and sequence analysis of a polygalacturonase-encoding gene from the phytopathogenic fungus *Sclerotinia sclerotiorum*. *Gene* 146 233–237. 10.1016/0378-1119(94)90298-4 8076824

[B45] Riou C. Freyssinet G. Fevre M. (1991). Production of cell wall-degrading enzymes by the phytopathogenic fungus *Sclerotinia sclerotiorum*. *Appl. Environ. Microbiol.* 57 1478–1484. 16348487 10.1128/aem.57.5.1478-1484.1991 PMC182972

[B46] Rollins J. A. (2003). The *Sclerotinia sclerotiorum pac1* gene is required for sclerotial development and virulence. *Mol. Plant Microbe Interact.* 16 785–795. 10.1094/MPMI.2003.16.9.785 12971602

[B47] Sella L. Gazzetti K. Faoro F. Odorizzi S. D’Ovidio R. Schäfer W. (2013). A *Fusarium graminearum* xylanase expressed during wheat infection is a necrotizing factor but is not essential for virulence. *Plant Physiol. Biochem.* 64 1–10. 10.1016/j.plaphy.2012.12.008 23337356

[B48] Tamura K. Peterson D. Peterson N. Stecher G. Nei M. Kumar S. (2011). MEGA5: molecular evolutionary genetics analysis using maximum likelihood, evolutionary distance, and maximum parsimony methods. *Mol. Biol. Evol.* 28 2731–2739. 10.1093/molbev/msr121 21546353 PMC3203626

[B49] ten Have A. Tenberge K. B. Benen J. A. Tudzynski P. Visser J. van Kan J. A. (2002). “The contribution of cell wall degrading enzymes to pathogenesis of fungal plant pathogens,” in *The Mycota, Agricultural Applications*, Vol. XI, ed. Kempken F. (Berlin: Springer Verlag), 341–358. 10.1007/978-3-662-03059-2_17

[B50] Wagner F. Kusserow H. Schäfer W. (2000). Cloning and targeted disruption of two polygalacturonase genes in *Penicillium olsonii*. *FEMS Microbiol. Lett.* 186 293–299. 10.1111/j.1574-6968.2000.tb09120.x 10802187

[B51] Waksman G. Keon J. P. R. Turner G. (1991). Purification and characterization of two endopolygalacturonases from *Sclerotinia sclerotiorum*. *Biochim. Biophys. Acta* 1073 43–48. 10.1016/0304-4165(91)90180-O 1991145

[B52] Wanjiru W. M. Kang Z. Buchenauer H. (2002). Importance of cell wall degrading enzymes produced by *Fusarium graminearum* during infection of wheat heads. *Eur. J. Plant Pathol.* 108 803–810. 10.1023/A:1020847216155

[B53] Willetts H. J. Bullock S. (1992). Developmental biology of sclerotia. *Mycol. Res.* 96 801–816. 10.1016/S0953-7562(09)81027-7

[B54] Wu B. M. Subbarao K. V. (2008). Effects of soil temperature, moisture, and burial depths on carpogenic germination of *Sclerotinia sclerotiorum* and *S. minor*. *Phytopathology* 98 1144–1152. 10.1094/PHYTO-98-10-1144 18943461

[B55] Wu S. C. Halley J. E. Luttig C. Fernekes L. M. Gutiérrez-Sanchez G. Darvill A. G. (2006). Identification of an endo-β-1, 4-D-xylanase from *Magnaporthe grisea* by gene knockout analysis, purification, and heterologous expression. *Appl. Environ. Microbiol.* 72 986–993. 10.1128/AEM.72.2.986-993.2006 16461639 PMC1392926

[B56] Wu S.-C. Ham K.-S. Darvill A. G. Albersheim P. (1997). Deletion of two *endo*-β-1, 4-xylanase genes reveals additional isozymes secreted by the rice blast fungus. *Mol. Plant Microbe Interact.* 10 700–708. 10.1094/MPMI.1997.10.6.700

[B57] Xiao X. Xie J. Cheng J. Li G. Yi X. Jiang D. (2014). Novel secretory protein Ss-Caf1 of the plant-pathogenic fungus *Sclerotinia sclerotiorum* is required for host penetration and normal sclerotial development. *Mol. Plant Microbe Interact.* 27 40–55. 10.1094/MPMI-05-13-0145-R 24299212

[B58] Yajima W. Liang Y. Kav N. N. V. (2009). Gene disruption of an arabinofuranosidase/β-xylosidase precursor decreases *Sclerotinia sclerotiorum* virulence on canola tissue. *Mol. Plant Microbe Interact.* 22 783–789. 10.1094/MPMI-22-7-0783 19522560

[B59] Young N. Ashford A. E. (1992). Changes during development in the permeability of sclerotia of *Sclerotinia minor* to an apoplastic tracer. *Protoplasma* 167 205–214. 10.1007/BF01403384

[B60] Yu Y. Xiao J. Yang Y. Bi C. Qing L. Tan W. (2015). *Ss-Bi1* encodes a putative BAX inhibitor-1 protein that is required for full virulence of *Sclerotinia sclerotiorum*. *Physiol. Mol. Plant Pathol.* 90 115–122. 10.1016/j.pmpp.2015.04.005

[B61] Zhou C. Bai J. Deng S. Wang J. Zhu J. Wu M. (2008). Cloning of a xylanase gene from *Aspergillus usamii* and its expression in *Escherichia coli*. *Bioresour. Technol.* 99 831–838. 10.1016/j.biortech.2007.01.035 17376674

[B62] Zhu W. Wei W. Fu Y. Cheng J. Xie J. Li G. (2013). A secretory protein of necrotrophic fungus *Sclerotinia sclerotiorum* that suppresses host resistance. *PLoS ONE* 8:e53901 10.1371/journal.pone.0053901 PMC3544710 23342034

