# Supplementary figures and images for: Disruption of the Gene Encoding Endo-β-1, 4-Xylanase Affects the Growth and Virulence of Sclerotinia sclerotiorum
Source: Front Microbiol. 2016 Nov 10;7:1787. doi: 10.3389/fmicb.2016.01787 (PMC5103160; doi:10.3389/fmicb.2016.01787)

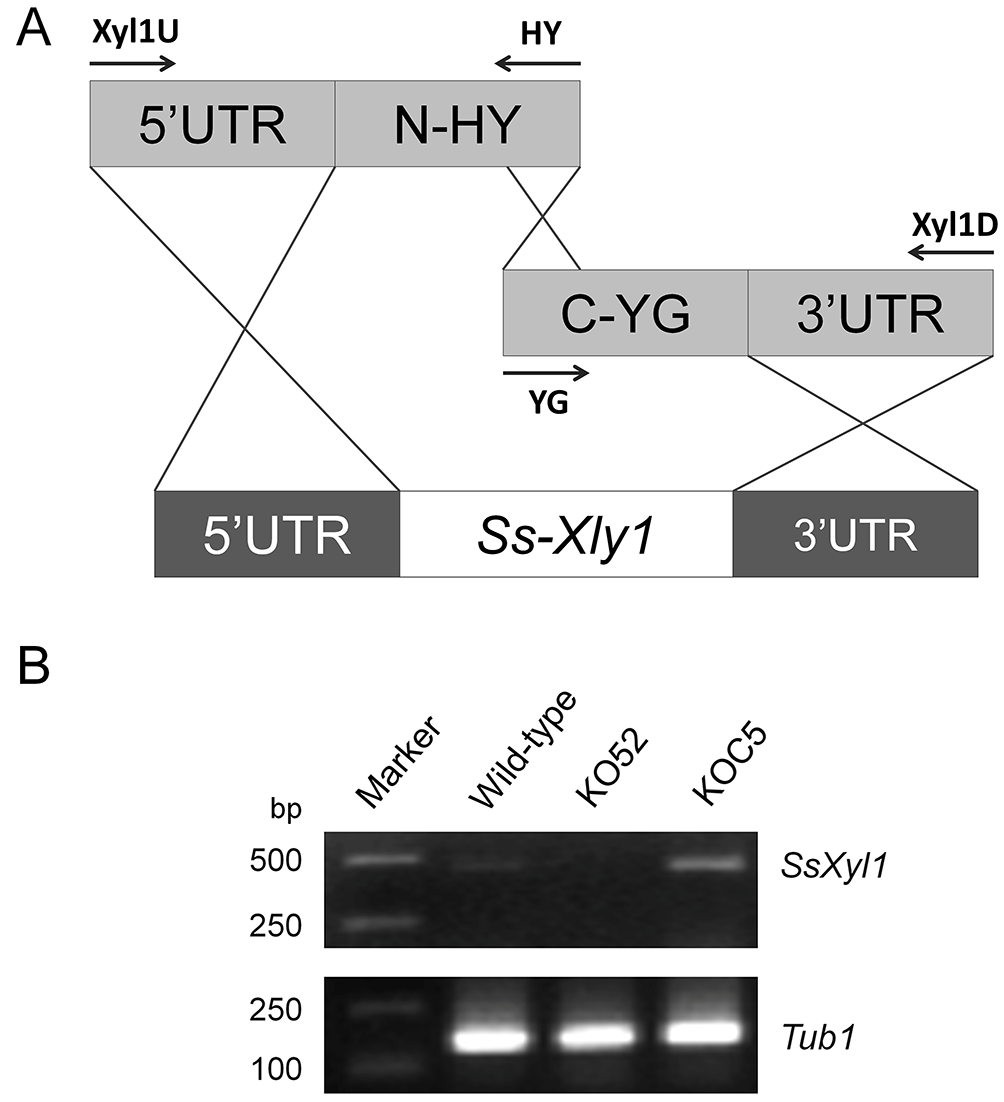

Supplement: FIGURE S1 — Disruption of the SsXyl1 gene function using a split marker strategy. (A) Schematic describing an overlapping hygromycin phosphotransferase gene (hph) marker for SsXyl1 disruption. The N-HY and C-YG imply 741 bp overlapping the 5′ and 3′fragments of hph gene, respectively. (B) RT-PCR analysis the expressions of SsXyl1 in the wild-type, gene deletion (KO52), and complemented (KOC5) strains. The expression of the Tub gene was the internal control. [file Image_1.TIF]
